# Psychometric properties of the Providers Survey in the Brazilian context of mental health: a validation study

**DOI:** 10.1590/1516-3180.2022.0444.R3.010623

**Published:** 2023-08-04

**Authors:** Mayara Karoline Silva Lacerda, Maria Aparecida Vieira, Fabrine Costa Marques, Juliana Pereira Alves, Matheus Mendes Pereira, Andreia Cristina Feitosa do Carmo, Mark Napoli Costa, Antônio Prates Caldeira, Cristina Andrade Sampaio

**Affiliations:** I MSc. Nurse and Professor, Bachelor in Nursing, Faculdade Prominas, Montes Claros (MG), Brazil.; II PhD. Nurse and Full Professor, Department of Nursing, Universidade Estadual de Montes Claros (UNIMONTES), Montes Claros (MG), Brazil.; III MSc. Nurse, Postgraduate Program in Health Sciences, Universidade Estadual de Montes Claros (UNIMONTES), Montes Claros (MG), Brazil.; IV MSc. Nurse, Postgraduate Program in Primary Health Care, Universidade Estadual de Montes Claros (UNIMONTES), Montes Claros (MG), Brazil.; V MSc. Nurse and Professor, Undergraduate Course in Nursing, Faculdade Prominas, Montes Claros (MG), Brazil.; VI MSc. Librarian, Universidade Federal de São Paulo (UNIFESP), São Paulo (SP), Brazil.; VII MD, MSc. Department of Psychiatry, Yale School of Medicine, Connecticut (CT), United States.; VIII MD, PhD. Professor, Department of Women's and Children's Health, Universidade Estadual de Montes Claros (UNIMONTES), Montes Claros (MG), Brazil.; XI PhD. Anthropologist and Professor, Department of Mental Health and Collective Health, Universidade Estadual de Montes Claros (UNIMONTES), Montes Claros (MG), Brazil.

**Keywords:** Validation study [publication type], Mental health recovery, Reproducibility of results, Data management, Health professionals, Psychometric properties, Instrument validation

## Abstract

**BACKGROUND:**

Precisely determining the aspects related to an instrument's validity and reliability measures allows for greater assurance of the quality of the results.

**OBJECTIVES:**

To analyze the psychometric properties of The Providers Survey in the Brazilian context of mental health services.

**DESIGN AND SETTING:**

The instrument validation study was conducted in Montes Claros, Minas Gerais, Brazil.

**METHODS:**

The validation study was conducted using the Consensus-based Standards for the Selection of Health Measurement Instruments checklist to analyze its validity and reliability.

**RESULTS:**

A committee of expert judges performed content validation after which the Content Validity Index was calculated. Construct validation took place through Exploratory Factor Analysis using the Kaiser-Meyer-Olkin Test criterion and Bartlett's Sphericity Test. Reliability was verified using test-retest reliability. The significance level adopted for the statistical tests was 5% (P < 0.05). The final instrument comprised 54 questions. The Content Validity Index was 97%. Exploratory Factor Analysis identified a Kaiser-Meyer-Olkin index of 0.901 and Bartlett's Sphericity Test with P < 0.001. We obtained a Cronbach's alpha coefficient of 0.95 and an intraclass correlation coefficient of 0.849.

**CONCLUSIONS:**

The Providers Survey, translated and adapted into Portuguese, was named the Work Assessment Instrument for the Recovery of Mental Health. It presented adequate psychometric properties for evaluating work-related practices for the recovery of psychosocial care network users.

## INTRODUCTION

The mental health policy in Brazil has shifted from a clinical model with an emphasis on reducing or removing mental health symptoms to a broader understanding based on an active, non-linear ongoing journey that involves rebuilding oneself and living a full and meaningful life. To this end, the concept of health is intimately disconnected from the absence of linked diseases, and harmonization exists between all conditioning factors and determinants of health, such as food, housing, leisure, safety, and work. The weight of these factors provides an individual with health from a biopsychosocial perspective.^1,2^

The inclusion of patients with mental disorders in daily and social activities began in Brazil in the 1970s and in the United States and in other European countries in the mid-1980s, and the use of the Recovery concept expanded. The expansion of this concept took place through the mobilization of users, family members, professionals, and managers in favor of actions that would provide an optimistic model of personal power to users of Mental Health services, thus consolidating a set of elements that empowered them to redirect their lives after being diagnosed with a mental disorder.^3^

One proposal for the implementation of Recovery for people with mental disorders is their insertion into the labor market, as work can act as a support component in the reinstatement of these individuals.^4,5^

Studies indicate that the inclusion of patients with mental disorders in the labor market has a positive economic, psychosocial, and clinical impact on their lives.^6,7^ Another important point is that employment correlates with short-term reductions in mental health costs.^8,9^

In 2016, in the State of Connecticut in the United States, The Providers Survey instrument was developed to verify the perception of job providers regarding the relationship between work and the recovery of people with mental disorders.^10^ There is a lack of evaluative instruments regarding the proposals of Recovery applied in the Brazilian cultural context of Mental Health services, despite its strength in the international scenario.

Precisely determining the aspects related to the instrument's validity and reliability measures allows for greater assurance of the quality of results. It is worth noting, however, that validity and reliability are not fixed measures and may vary according to the population, type of study, and its purpose.^11^

For this study, this proposal was adopted to validate The Providers Survey instrument, based on an analysis of its psychometric properties, given that its cross-cultural adaptation was conducted in a previous study. This study contributes to clinical practice in the field of mental health by stimulating research on the perceptions of health professionals regarding the relationship between work and the recovery of people with mental disorders.^12^

## OBJECTIVES

This study aimed to analyze the psychometric properties of The Providers Survey instrument in the Brazilian context of mental health services through validation using the Consensus-Based Standards for the Selection of Health Measurement Instruments checklist for the analysis of content, construct validity, and reliability.

## METHODS

This instrument validation study aims to describe the psychometric properties of The Providers Survey in the Brazilian context of Mental Health services after cross-cultural adaptation.

For validation, we used the Consensus-Based Standards for the Selection of Health Measurement Instruments checklist to analyze the characteristics of validity and reliability.^13^ The validity measure was verified by content validation through the evaluation of a committee of expert judges, followed by the calculation of the Content Validity Index, which was validated using Exploratory Factor Analysis. The reliability measure was verified from the analysis of Internal Consistency by Cronbach's alpha coefficient and the test-retest stability by the Intraclass Correlation Coefficient. Data were tabulated and analyzed using SPSS version 22.0 for Windows program (IBM, Armonk, New York, United States). Validity and reliability characteristics are relevant domains for validating measurement instruments.^14,15^

This project was approved by the Research Ethics Committee of the Universidade Estadual de Montes Claros (UNIMONTES) under Opinion No. 2,398,868 (Montes Claros, November 25, 2017). All participants agreed to participate in the study by signing the Free and Informed Consent Term form.

The Providers Survey instrument's original version was developed in Connecticut, the United States, and has 85 questions distributed in four domains. After cross-cultural adaptation for use in Brazil, the instrument presented 65 questions distributed in three domains: Domain I, with 26 questions referring to important aspects of working with clients with mental disorders; Domain II, with 16 questions related to factors that allow people with mental disorders to obtain and maintain a job; and Domain III, with 23 questions related to factors that promote the recovery of people with mental disorders.^12^

After the cross-cultural adaptation, the psychometric properties of the instrument were verified. Subsequently, the instrument had 54 questions, with 24 questions in the first part of the instrument related to the work of health professionals with users in mental suffering, 14 questions referring to the components that allow users in mental suffering to obtain and maintain their jobs, and 16 questions referring to the importance of the components to promote the restoration/recovery of users in mental suffering.

Data collection for the assessment of the instrument's psychometric properties after cross-cultural adaptation was conducted between December 2019 and January 2020.

## RESULTS

For content validation, 10 Mental Health specialists, all with knowledge about Recovery, participated in this study and comprised a committee of expert judges/experts.

We performed an Exploratory Factor Analysis to validate this construct. At this stage, 496 questionnaires were sent, and 364 responses were returned. Of the total questionnaires answered, eight professionals refused to participate in the research and 38 questionnaires were not fully answered, leading to a loss rate of 26.6% (n = 132). Finally, 318 valid questionnaires were obtained. After validating the instrument, we verified its reliability through Internal Consistency analysis and its stability through test-retest.

### Content validation

The content validation stage of the Providers Survey instrument included the participation of a committee of 10 specialist professionals with different activities in the components of the Psychosocial Care Network in the city of Montes Claros, MG, Brazil, who had knowledge of mental health recovery. Of these professionals, two were doctors, one was a nurse, one was a dentist, and six were psychologists, as described in Table 1.

**Table 1:** Characterization of the members of the expert judges committee, Montes Claros, 2019

Specialist	Academic education	Experience in the field of Mental Health
1	Psychologist. Mental Health Specialist.	Preceptor of the Multiprofessional Residency in Family Health. Works in Primary Care. She served as a psychologist in Psychosocial Care Center.
2	Psychologist. Family Health Specialist.	Preceptor of the Multiprofessional Residency in Family Health. Works in Primary Care. Worked as a Psychosocial Care Center.
3	Psychologist. Family Health Specialist.	Preceptor of the Multiprofessional Residency in Family Health. Works in Primary Care.
4	Psychologist. Mental Health Specialist.	Preceptor of the Multiprofessional Residency in Family Health and Mental Health. Works in Primary Care. Worked as a Psychosocial Care Center.
5	Nurse. Family Health Specialist. Master in Primary Health Care.	Preceptor of the Multiprofessional Residency in Family Health. Works in Primary Care service.
6	Doctor. Specialist in Psychiatry and Psychotherapy.	Coordinator of the Residency in Psychiatry. Operates in the private network. Professor at the Department of Medicine at the State University of Montes Claros.
7	Psychologist. Doctor in Psychology. Master in Public Health.	Professor at the Department of Mental Health and Collective Health at the State University of Montes Claros.
8	Psychologist. Mental Health Specialist.	Works in Primary Care. Worked as an intern at Psychosocial Care Center.
9	Dentist surgeon. Family Health Specialist.	Preceptor of the Multiprofessional Residency in Family Health. Works in Primary Care service.
10	Doctor. Specialist in Family and Community Medicine.	Preceptor of the Residency in Family and Community Medicine. Works in Primary Care service.

Source: Prepared by the authors.

We sent a questionnaire containing The Providers Survey with 74 items to be evaluated by the expert committee. The content of this instrument refers to the Informed Consent Term form; participant identification data; type of service; position; working time; instrument title; and 26 questions related to Domain I, 16 questions related to Domain II, and 23 questions related to Domain III.

To verify the clarity, scope of the items, and the general structure and layout of the instrument, this questionnaire included a specific field related to each item so that each specialist could put their considerations regarding the item evaluated for further adequacy. Throughout the evaluation process, the responsible team was able to clarify doubts and possible queries.

To conduct the aforementioned assessment of the instrument's items, we used a 4-point Likert scale, in which each item of the instrument received the following classification: 1) being a non-equivalent item; 2) a slightly equivalent item that needs many revisions; 3) an equivalent item that requires few revisions; and 4 a fully equivalent item. Instrument items with a score of 1 or 2 were excluded.

After this step, the instrument presented 69 items, as two questions from the first domain, two questions from the second domain, and one question from the third domain were excluded.

After the subjective assessment by the group of experts, the Content Validity Index was calculated. The 74 items were analyzed by 10 committee participants, totaling 740 responses, with 10 responses referring to each item of the instrument. From the sum of all responses classified as 3 or 4, followed by dividing by the total number of responses, we obtained a Content Validity Index of 97%.

### Construct validation

Construct validity, also known as factor validity, is considered one of the most important measures of instrument validity in research and aims to verify whether the items of the instrument have a reliable and adequate representation of the construct to be measured.

At this stage, 318 health professionals with higher education working in cities included in the Regional Health Management of Montes Claros, located in the north of the state of Minas Gerais, Brazil, participated as respondents, as described in Table 2.

**Table 2:** Characterization of professionals who participated in construct validation, Montes Claros, MG, Brazil, 2019 (n = 318)

Variables		n	%
**Age (years)**			
	18–24	13	4.1
	25–34	162	50.9
	35–44	108	34.0
	45–54	28	8.8
	55–64	6	1.9
	65–74	1	0.3
**Total**		**318**	**100.0**
**Gender**			
	Man	71	22.3
	Woman	247	77.7
**Total**		**318**	**100.0**
**County**			
	Montes Claros	281	88.4
	Bocaiuva	3	0.9
	Janaúba	2	0.6
	Salinas	5	1.6
	Monte Azul	2	0.6
	Rather not answer	25	7.9
**Total**		**318**	**100.0**
**Occupation area**			
	Service professionals not specialized in Mental Health	269	84.6
	Professionals of specialized services in Mental Health	46	14.5
	Rather not answer	3	0.9
**Total**		**318**	**100.0**
**Office**			
	Social worker	12	3.8
	Dental surgeon	63	19.8
	Coordinator/Manager	15	4.7
	Nurse specialist in mental health	9	2.8
	Nurse not specialist in mental health	119	37.4
	Pharmaceutical	2	0.6
	Physician (not psychiatrist)	62	19.5
	Psychiatrist	2	0.6
	Workshop monitor	1	0.3
	Psychologist	29	9.1
	Occupational therapist	3	0.9
	Rather not answer	1	0.3
**Total**		**318**	**100.0**
**Working time (years)**			
	Less than 1	26	8.2
	1–2	69	21.7
	3–5	84	26.4
	6–9	64	20.1
	10 or more	75	23.6
**Total**		**318**	**100.0**

Source: Prepared by the authors.

The instrument version that also contained the ICF was sent via WhatsApp to 318 participants. The responses were consolidated in the Qualtrics Survey Software Platform, which is an online survey platform, from which the data were sent directly to Microsoft Excel 2016 (version 16.0) for Windows (Microsoft Corporation, Redmond, Washington, United States) and then exported to SPSS version 22.0, for Windows (National Opinion Research Center, Chicago, Illinois, United States).

The initial Exploratory Factor Analysis procedures indicated a Kaiser-Meyer-Olkin index of 0.901 and Bartlett's test, with a significance level of P < 0.001. The model was adjusted using principal component analysis. Varimax orthogonal rotation was used because it treats domains as independent. In the analysis of commonalities, all variables with factor loadings greater than 0.5 were maintained for matrix rotation. Six questions were eliminated because they had values lower than 0.5, as shown in Table 3. The factor loadings for each variable allowed for the identification of each variable with the respective factor. The three main factors/components of the instrument represent 49.1% of the total cumulative explained variance. After the construct validation stage, the instrument presented 54 questions distributed across three domains.

**Table 3:** Rotating component matrix elaborated in the construct validation stage, Montes Claros, Minas Gerais, Brazil, 2019

Variables	Components		
	1	2	3
Hosting the user, their family, and/or companions	0.5	0.1	0.0
Recording a complete personal and family anamnesis/history	0.7	0.1	0.1
Conducting case follow-up	0.6	0.3	0.0
Providing a reference technician for case management	0.5	0.1	0.1
Offering counseling and/or guidance and/or psychotherapy or other psychological interventions	0.6	0.1	-0.1
Participating in the formulation of the diagnosis	0.6	0.1	-0.1
Prescribing and/or administering medication	0.5	0.0	0.1
Monitoring medication adherence	0.6	0.1	0.1
Providing guidance on the use of medications and the effect on the user's psychological condition for the user, their family members and/or companions	0.7	0.1	0.1
Identifying and addressing stigma and discrimination/prejudice	0.6	0.0	0.2
Referring users to medical care and assistance	0.6	0.2	0.0
Referring users to social support services to search for work/employment (legal associations and public services)	0.7	0.1	0.2
Providing job search support	0.6	0.0	0.3
Referring users to the treatment of alcoholism and/or the use of psychoactive substances	0.7	0.3	0.0
Providing treatment for alcoholism and/or the use of other psychoactive substances	0.6	0.2	0.0
Referring users to the housing support service	0.6	0.0	0.2
Providing housing search support	0.5	0.0	0.2
Referring users to socialization activities and recreational/leisure support	0.7	0.0	0.1
Providing socialization activities and recreational/leisure support	0.6	0.0	0.2
Referring users to legal support	0.5	-0.1	0.2
Involving family members in supporting users in mental distress	0.7	0.1	0.0
Providing peer support and guidance and/or therapeutic companion	0.7	0.1	0.1
Providing guidance on the self-care and well-being of users	0.7	0.3	0.0
Addressing issues related to the various forms of violence and/or other forms rights violations	0.7	0.1	0.2
Setting a goal for getting a job	0.2	0.1	0.6
Including users in work/employment support programs, regardless of the severity of their symptoms or other underlying difficulties	0.1	0.2	0.6
Conducting a job search as soon as the person shows interest in working	0.2	0.1	0.7
Integrating vocational support with clinical care for the user in an individualized way	0.2	0.1	0.8
Being aware of users’ preferences regarding a job/occupation	0.2	0.1	0.7
Identifying and addressing cases of discrimination at work/employment	0.2	0.1	0.7
Encouraging the person to seek employment	0.2	0.3	0.5
Identifying and addressing negative internalized views of themselves that make people believe they are incapable of working	0.2	0.3	0.6
Involving family members in supporting the user's efforts to seek or maintain employment	0.3	0.4	0.5
Engaging peers to support the user's efforts to seek or maintain work/job	0.3	0.3	0.6
Recognizing work/employment as an important need in the restoration of the user	0.2	0.4	0.6
Recognizing the job as a source of stress that should be avoided	-0.1	0.0	0.6
Recognizing employment as offering a valuable social role or as an important source of identity reinforcement	0.2	0.3	0.5
Recognizing employment as a factor that can increase the risk of relapse/crisis of users in mental suffering	-0.1	0.0	0.6
Being connected to something that goes beyond oneself (e.g., spirituality/religiosity)	0.0	0.3	0.3
Being hopeful	0.0	0.7	0.1
Having a life project	0.2	0.7	0.0
Having stable housing/place	0.1	0.7	0.2
Being abstinent from drugs and alcohol	0.1	0.5	0.2
Being employed in formal or informal work	0.1	0.4	0.4
Eliminating all psychiatric symptoms	0.0	0.2	0.4
Having family support	0.1	0.7	0.0
Having friends or people to trust and/or partner or spouse	0.2	0.7	0.0
Adhering to prescribed treatments	0.1	0.7	0.0
Adapting to psychiatric symptoms	0.0	0.2	0.4
Having a sense of belonging in the community and valuing their cultural and social identity	0.2	0.7	0.2
Taking control of one's own life/autonomy	0.1	0.6	0.3
Having something that gives meaning/meaning to life	0.1	0.6	0.2
Having quality medical care and/or multi-professional assistance	0.1	0.7	0.1
Believing in oneself as a capable person	0.1	0.8	0.0
Being financially independent	0.0	0.4	0.3
Participating in recreational/leisure social activities	0.1	0.6	0.2
Having a long period of stability (i.e., no crises)	0.0	0.5	0.1
Eating healthy and practicing physical activity	0.1	0.6	0.2
Questioning and rejecting social stereotypes of users in mental distress (e.g., “patient with mental illness” or addict)	0.0	0.3	0.3
Being valued for their activities in the community	0.1	0.7	0.2

Extraction method: Principal component analysis.

Rotation method: Varimax with Kaiser normalization.

### Reliability

The reliability of the instrument was verified after content and construct validation. We verified the Internal Consistency using the α-Cronbach's coefficient and the stability using the test-retest using the Intraclass Correlation Coefficient.

For the stability analysis, we used the test-retest with a sample of 51 respondents, with an average interval of 12 days after the test. The Intraclass Correlation Coefficient to verify the stability of the instrument which presented a value of 0.849 using the Pearson correlation test (P < 0.001), and the verification of the internal consistency returned an α-Cronbach of 0.95.

The significance level adopted for the statistical tests was 5% (P < 0.05). All statistical analyses were performed using IBM SPSS statistical package software, version 22.0, for Windows (National Opinion Research Center, Chicago, Illinois, United States). The final version of this instrument is presented in Table 4.

**Table 4:** Final Version of the Work Assessment Instrument for Recovery in Mental Health – (IATRE-SM), Montes Claros, Minas Gearis, Brazil, 2019 (Brazilian version of The Providers Survey instrument)

PART 1						
Please indicate how important the following activities are in your work with mentally ill users. Where: 1-Not important, 2-Somewhat important, 3-Not at all and not very important, 4-Important, 5-Very important, 6-Not part of my job).						
**DOMAIN**	**1**	**2**	**3**	**4**	**5**	**6**
Hosting the user, their family, and/or companions						
Recording a complete personal and family anamnesis/history						
Conducting case follow-up						
Providing a reference technician for case management						
Offering counseling and/or guidance or psychotherapy/other psychological interventions						
Participating in the formulation of the diagnosis						
Prescribing and/or administering medication						
Monitoring medication adherence						
Guiding the use of medications and the effect on the user's psychic condition for the user, their family members, and/or companions						
Identifying and addressing stigma and discrimination/prejudice						
Referring users for medical attention and care						
Referring users to social support services to search for work/employment (legal associations and public services)						
Providing job search support						
Referring users for treatment of alcoholism and/or the use of psychoactive substances						
Providing treatment for alcoholism and/or the use of other psychoactive substances						
Referring users to the housing support service						
Providing housing search support						
Referring users to socialization activities and recreational/leisure support						
Providing socialization activities and recreational/leisure support						
Referring users to legal support						
Involving family members in supporting users in mental distress						
Providing peer support and guidance and/or a therapeutic companion						
Providing guidance on the self-care and well-being of users						
Addressing issues related to forms of violence or forms rights violations.						
**PART 2**						
**Please indicate how important the following components are to enabling users in mental distress to obtain and keep their jobs. Where: 1-Not important, 2-Somewhat important, 3-Not at all and not very important, 4-Important, 5-Very important, 6-I prefer not to answer.**						
**DOMAIN**	**1**	**2**	**3**	**4**	**5**	**6**
Setting a goal for getting a job						
Including users in work/employment support programs, regardless of the severity of their symptoms or other underlying difficulties						
Conducting a job search as soon as the person shows interest in working						
Integrating vocational support with clinical care in an individualized way						
Being aware of users’ preferences regarding a job/occupation						
Identifying and addressing cases of discrimination at work/employment						
Encouraging the person to seek employment						
Identifying and addressing negative internalized views of themselves that make people believe they are incapable of working						
Involving the family in supporting the user's efforts to seek or maintain employment						
Engaging peers in supporting the user's efforts to seek or maintain work/job						
Recognizing the work/job as important in the recovery of the user						
Recognizing the job as a source of stress that should be avoided						
Recognizing employment as offering a valuable social role or as an important source of identity reinforcement						
Recognizing employment as a risk factor for relapse/crisis of users in mental suffering						
**PART 3**						
**Indicate the degree of importance of the following components to promote the restoration/recovery of users in mental suffering. Where: 1-Not important, 2-Somewhat important, 3-Not at all and not very important, 4-Important, 5-Very important, 6-I prefer not to answer.**						
**DOMAIN**	**1**	**2**	**3**	**4**	**5**	**6**
Being hopeful						
Having a life project						
Having stable housing/place						
Being abstinent from drugs and alcohol						
Having family support						
Having friends or people to trust and/or partner or spouse						
Adhering to prescribed treatments						
Having a sense of belonging in the community and value their cultural and social identity						
Taking control of their own life/autonomy						
Having something that gives meaning to life						
Having quality medical care and/or multi-professional assistance						
Believing in oneself as a capable person						
Participating in recreational/leisure social activities						
Having a long period of stability (i.e., no crises)						
Eating healthy and practice physical activity						
Being valued for their activities in the community						

After analyzing the psychometric properties of The Providers Survey instrument, it is clear that such measures may vary according to changes in the study population and context presented. It is desirable that new studies be conducted in several Brazilian regions to verify the attitudes and actions of professionals focused on incentive practices and approaches aimed at the recovery of the Psychosocial Care Network service users.

## DISCUSSION

We used a committee of expert judges to evaluate the constructs; of this committee, at least five were specialists in the area.^16^ Based on the judgment of experts in the field, we verified the degree of equivalence of each item of the instrument under analysis to measure the degree of relevance of each item of the instrument in a given construct.

After the subjective assessment performed by the group of experts, we calculated the Content Validity Index, which measures the percentage of judges who agreed on the aspects of each item of the instrument.^14,15^ Instrument items that received a score of 1 or 2 were excluded.^13^ An acceptable Content Validity Index must be at least 0.80, and preferably greater than 0.90.^12,14,15,17,18^

For construct validation, we used the Factor Analysis strategy, considered one of the most important measures of instrument validity in research that aim to verify if the items of a given instrument are a reliable and adequate representation of the construct to be measured.^14,15^

Following the recommendations regarding the constitution of the sample to conduct a Factor Analysis, we aimed for the participation of at least 100 respondents, with 5–10 respondents being ideal for each item of the questionnaire.^11,19–21^

To assess the factorial structure of the instrument, we used the Kaiser-Meyer-Olkin index and Bartlett's Sphericity Test with a significance level of P < 0.001, which indicates the suitability of factor analysis in the process.^22^

After verifying the suitability of the factor analysis, we rotated the matrix to separate the variables between factors. The varimax rotation method is the most commonly used method in research of this nature, as it treats domains as independent allowing us to exclude questions with a factor loading < 0.5. Studies recommend that the Principal Components should have eigenvalues > 1.^23–25^

To assess the reliability of an instrument, we used the internal consistency based on Cronbach's alpha coefficient and the stability based on the test-retest using the Intraclass Correlation Coefficient via the Pearson correlation test.^14,15^ The test-retest, which allows the reproduction of a result in time and space, provides homogeneity and equivalence between different respondents.^13,16^ Test-retest reliability tends to decrease when the time interval is prolonged, and for this reason, we conducted a verification within an interval of 10 to 14 days between the test and retest on a sample of at least 15% of the participants or a minimum of 50 respondents.^26,27^

For the verification of internal consistency, values above 0.70 for the α-Cronbach are accepted as adequate. As for the Intraclass Correlation Coefficient, values between 0.6 and 0.79 indicate a substantial correlation and values greater than or equal to 0.80 indicate an almost complete correlation.^14,15,26,27^

It is noteworthy that the present study followed all the recommendations proposed to verify the psychometric properties of measurement instruments considering the context of mental health in Brazil through an approach to health professionals regarding the importance of work as an important component in individuals’ mental health recovery.

This study is limited by the variability in the context and organization of The Political Action Network for Sustainability in each region of Brazil, resulting in different results in different contexts. Precisely determining the aspects related to the instrument's validity and reliability measures allows for greater assurance of the quality of results. However, it is worth clarifying that validity and reliability are not fixed measures and may vary according to the population, type of study, and purpose.

## CONCLUSION

Through the values obtained in the validation process of The Providers Survey instrument using the Consensus-Based Standards for the Selection of Health Measurement Instruments checklist, we found that the Brazilian version of the instrument had content validity, construct validity, and reliability, which was verified by internal consistency and stability. Therefore, it is an instrument capable of exploring the phenomenon to be studied, and its items reliably and adequately represent the measured construct. All the instrument's parts measure the same characteristic, which guarantees its reliability, and confers homogeneity among the different respondents.

The instrument developed instrument was named the Work Assessment Instrument for Recovery in Mental Health (IATRE-SM). From its use, it is expected that mental health services in Brazil will be guided by the Recovery concept to prepare users so that they can face society and engage in the recovery process. It is also expected that users will be perceived as individuals capable of integrating into society and exercising their autonomy, assuming an active role in the community. Based on the results of this study, we encourage research on the perceptions of health professionals regarding the relationship between work and recovery for people with mental disorders.
